# A Distinct Microbiome Signature in Posttreatment Lyme Disease Patients

**DOI:** 10.1128/mBio.02310-20

**Published:** 2020-09-29

**Authors:** Madeleine Morrissette, Norman Pitt, Antonio González, Philip Strandwitz, Mariaelena Caboni, Alison W. Rebman, Rob Knight, Anthony D’Onofrio, John N. Aucott, Mark J. Soloski, Kim Lewis

**Affiliations:** a Antimicrobial Discovery Center, Department of Biology, Northeastern University, Boston, Massachusetts, USA; b Department of Pediatrics, University of California San Diego, La Jolla, California, USA; c Lyme Disease Research Center, Division of Rheumatology, Department of Medicine, Johns Hopkins University School of Medicine, Baltimore, Maryland, USA; d Department of Computer Science and Engineering, University of California, San Diego, La Jolla, California, USA; e Center for Microbiome Innovation, University of California, San Diego, La Jolla, California, USA; Harvard Medical School

**Keywords:** Lyme disease, diagnostics, microbial communities, microflora, tick-borne pathogens

## Abstract

Lyme disease is the most common vector-borne disease in the United States, with an estimated incidence of 300,000 infections annually. Antibiotic intervention cures Lyme disease in the majority of cases; however, 10 to 20% of patients develop posttreatment Lyme disease syndrome (PTLDS), a debilitating condition characterized by chronic fatigue, pain, and cognitive difficulties. The underlying mechanism responsible for PTLDS symptoms, as well as a reliable diagnostic tool, has remained elusive. We reasoned that the gut microbiome may play an important role in PTLDS given that the symptoms overlap considerably with conditions in which a dysbiotic microbiome has been observed, including mood, cognition, and autoimmune disorders. Analysis of sequencing data from a rigorously curated cohort of patients with PTLDS revealed a gut microbiome signature distinct from that of healthy control subjects, as well as from that of intensive care unit (ICU) patients. Notably, microbiome sequencing data alone were indicative of PTLDS, which presents a potential, novel diagnostic tool for PTLDS.

## Abstract (precis)

Most patients with acute Lyme disease are cured with antibiotic intervention, but 10 to 20% endure debilitating symptoms such as fatigue, neurological complications, and myalgias after treatment, a condition known as posttreatment Lyme disease syndrome (PTLDS). The etiology of PTLDS is not understood, and objective diagnostic tools are lacking. PTLDS symptoms overlap several diseases in which patients exhibit alterations in their microbiome. We found that patients with PTLDS have a distinct microbiome signature, allowing for an accurate classification of over 80% of analyzed cases. The signature is characterized by an increase in *Blautia*, a decrease in *Bacteroides*, and other changes. Importantly, this signature supports the validity of PTLDS and is the first potential biological diagnostic tool for the disease.

## INTRODUCTION

Lyme disease, a tick-borne infection caused by Borrelia burgdorferi, affects approximately 300,000 people annually in the United States (1, 2). The symptoms of acute Lyme disease are highly variable, and when untreated, it can progress in severity over time from malaise and flu-like symptoms to neurological disorders, cardiac complications, and, in late stages, arthritis (3). Antibiotic intervention typically cures Lyme disease; however, approximately 10 to 20% of Lyme patients develop posttreatment Lyme disease syndrome (PTLDS) with symptoms, including myalgias, chronic fatigue, and cognitive difficulties for more than 6 months after completion of antibiotic treatment (4–8). The etiopathology of PTLDS is unknown, but it presents with symptoms that overlap those of other diseases, including chronic fatigue syndrome (5, 9), depression, fibromyalgia (5), and multiple sclerosis (10).

Along with unknown etiopathology and a diverse range of symptoms, diagnosing PTLDS remains challenging. Although a clinical case definition proposed by the Infectious Diseases Society of America (IDSA) in 2006 has served as a specific research tool, there is no biological method to diagnose PTLDS (11). While clinical biomarkers associated with PTLDS have been observed, a suitable diagnostic method and therapy remain elusive. A positron emission tomography (PET) brain imaging study among patients with PTLDS demonstrated elevated microglial activation compared to that of controls, congruent with localized inflammation (12). Additional research has shown that a greater B. burgdorferi-specific plasmablast response prior to treatment favors a resolution of symptoms versus the development of PTLDS, which indicates that even before treatment, a patient’s immunological landscape plays an important role in the development of PTLDS (13). Compared to healthy controls, patients with PTLDS have significantly elevated expression of interferon alpha, greater antibody reactivity to brain antigens (14), increased levels of the chemokine CCL19 (15) and the cytokine interleukin 23 (IL-23) (16), and a decrease in the CD57 lymphocyte subset (17, 18). Furthermore, patients have a higher risk of developing new-onset autoimmune joint diseases after a Lyme erythema migrans rash (19). Therefore, while the etiopathology is still unknown, these markers indicate biological abnormalities among patients with PTLDS.

The microbiome has been implicated in many diseases with symptoms that overlap those of PTLDS, including autoimmune diseases. The pathogenesis of autoimmune disease is affected by environmental (20) and genetic (21) factors, as well as the gut microbiome (22, 23). The gut microbiome plays an important role in human health and has been shown to strongly influence host metabolism (24, 25) and the immune (22, 26) and nervous (27) systems, as well as provide crucial colonization resistance against a range of intestinal pathogens (28, 29). Further, microbiome compositional changes can alter immune tolerance (22, 23). For instance, members of the intestinal microbiota have been characterized as contributing to the development of the long-term sequelae of acute infection events upon disruption of tissue and immune homeostasis (30). Studies have found the microbiome to be on par with and often superior to the human genome in predicting disease states (31, 32). Indeed, many microbiome-wide association studies have established correlation, and sometimes causation, of the gut microbiome in diseases such as multiple sclerosis (33, 34), rheumatoid arthritis (35), and systemic lupus (36). Patients with PTLDS often undergo extensive antibiotic treatment (5) which likely causes adverse alterations to their microbiomes. A potential parallel exists in autoimmune disease, in which antibiotic use has been linked to an increase in disease frequency because of the dramatic impact antibiotics have on the microbiome (37).

Since PTLDS symptoms present similarly to diseases in which the microbiome is implicated, we reasoned that the same may be true for the gut microbiome of patients with PTLDS. We analyzed the gut microbiome of subjects with PTLDS from the John Hopkins Lyme Disease Research Center’s Study of Lyme Immunology and Clinical Endpoints (SLICE) cohorts, specifically drawing from a cross-sectional cohort of patients meeting the IDSA-proposed case definition for PTLDS. We performed 16s rRNA gene sequencing on stool samples from this cohort and report a gut microbiome signature associated with PTLDS. These data present a novel biomarker and potential diagnostic tool for PTLDS, as well as suggest a therapeutic avenue for PTLDS.

## RESULTS

### Curation of the PTLDS and control cohorts.

Fecal samples were collected from 87 patients with well-defined PTLDS in the SLICE cohort. Patients had medical record documentation of prior Lyme disease which met the CDC surveillance case definitions for definitive or probable Lyme disease and current nonspecific, patient-reported symptoms meeting an operationalized case definition for PTLDS with symptoms leading to functional impairment (8). Patients had all received appropriate antibiotic treatment at the time of their initial diagnosis of Lyme disease, and many had received subsequent antibiotics for treatment of persistent symptoms. The median time from Lyme disease symptom onset to the study visit was 1.1 years (interquartile range [IQR], 0.5 years to 3.3 years), and participants reported taking a median of 56 days (IQR, 30 days to 84 days) of antibiotics during that interval. Eight (9.2%) reported currently taking antibiotics at the time of the study visit. The mean age of this cohort sample was 48.3 years (standard deviation [SD], 14.7), and 36 (41.4%) of the subjects were female.

The healthy control cohort consisted of fecal samples collected from 17 healthy donors at Northeastern University, as well as 152 donors from a previously identified healthy subset of the American Gut (38). To control for the generally high levels of antibiotic use that could alter the microbiome in patients with PTLDS, a previously curated cohort of 123 samples of intensive care unit (ICU) patients from two time points (39) was also used as a control.

**FIG 1:**
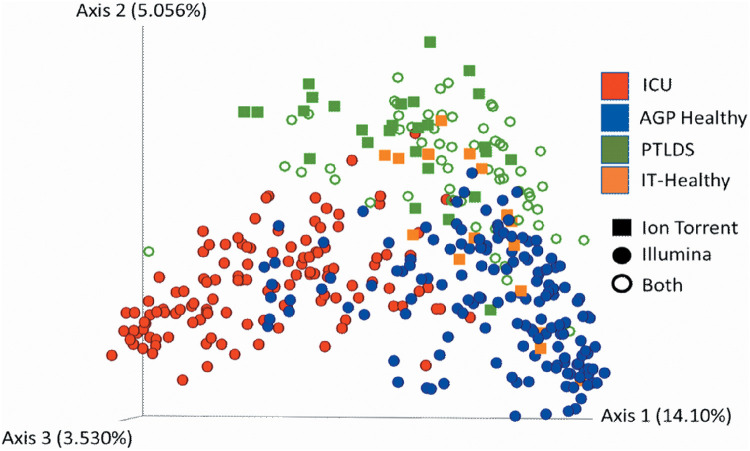
Principal-coordinate analysis of unweighted UniFrac distances of 16S rRNA genes. All samples from patients in the ICU, those with PTLDS, and the IT-Healthy and AGP Healthy cohorts are represented. The sequencing platform, Illumina, Ion Torrent, or both, used for each sample is indicated by shape.

To analyze the gut microbiome composition, 16S rRNA gene sequencing was performed using the Illumina and Ion Torrent platforms; the dual-platform approach did not have a measurable effect on the data (Fig. 1). The sequences, combined with the control sequencing data, were processed using Qiita (40) and Quantitative Insights into Microbial Ecology 2 (QIIME2) (41). Closed-reference operational taxonomic units (OTUs), a common designation used instead of “species” or “genus,” were generated (97% identity) and analyzed. Closed-reference picking was performed because it allowed for increased sample size of the PTLDS cohort due to samples being processed in different platforms, but the conclusions of this study do not differ from an analysis using a subset of samples sequenced by Illumina technology and processed with Deblur to generate amplicon sequence variants (42) (see Fig. S1 in the supplemental material).

10.1128/mBio.02310-20.1 FIG S1 (A) Receiver operating characteristic curve evaluating the ability to distinguish the Illumina sequencing data subset of the PTLDS from healthy and ICU controls based on the fecal microbiome. Sequences were processed using Deblur (A. Amir, D. McDonald, J. A. Navas-Molina, E. Kopylova, et al., mSystems 2:e00191-16, 2017, https://doi.org/10.1128/mSystems.00191-16) and amplicon sequence variants (ASV) were generated. AUC values are reported. The gray dashed line represents the null model. (B) The identity and corresponding confidence of the taxon classification of the 5 most important features for classifying PTLDS from healthy and ICU controls based on the fecal microbiome as designated by the most specific taxonomy that could be confidently identified. Download FIG S1, TIF file, 0.4 MB. Copyright © 2020 Morrissette et al. 2020 Morrissette et al. This content is distributed under the terms of the Creative Commons Attribution 4.0 International license.

### Sample classification of PTLDS, ICU, and healthy fecal microbiomes.

**FIG 2:**
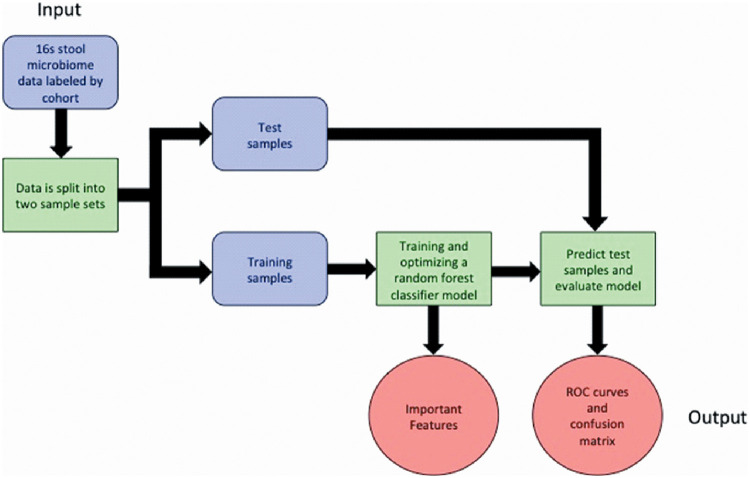
A simplified flow chart of the Qiime2 classifier model pipeline used to analyze the microbiome data.

**FIG 3:**
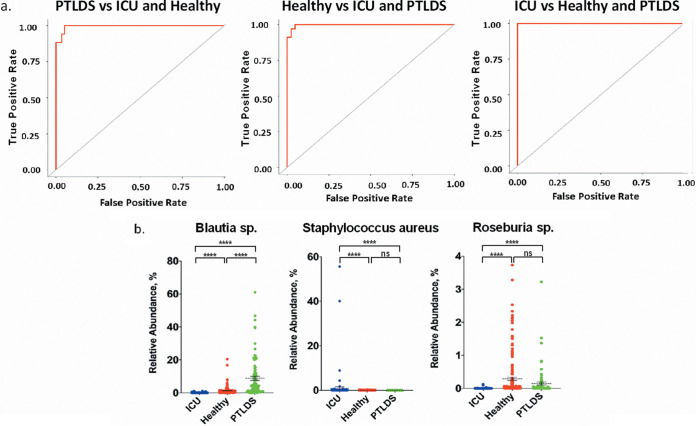
(a) Receiver operating characteristic curve evaluating the ability of a random-forest classifier model to classify PTLDS, healthy, and ICU controls based on the fecal microbiome determined by 16S rRNA gene sequencing. Rounded area under the ROC curve values were 1.00 for all cohorts. Gray lines represent the null model or random chance. (b) Relative abundance plots of the 5 most important features (OTUs) for classification of PTLDS, healthy, and ICU controls based on the fecal microbiome. The first, third, and fifth ranked most important features were *Blautia* species (OTU IDs 4474380, 4465907, and 4327141); the relative abundance of the *Blautia* spp. were combined for clarity. S. aureus (OTU ID 446058) and *Roseburia* sp. (OTU ID 4481427) were the second and fourth most important features, respectively. Bars represent the mean relative abundance plus or minus the standard error of the mean. Statistical significance was determined using Kruskal-Wallis (nonparametric) test followed by Dunn’s multiple comparison. ****, *P* value < 0.0001. ns, not significant.

We evaluated the ability of the fecal microbiome to distinguish PTLDS, ICU, and healthy cohorts using a supervised-learning random-forest classifier model to classify sample cohorts (43) (Fig. 2). Receiver operating characteristic (ROC) analysis was used to evaluate the accuracy of the model’s classifications (44). The model’s performance was quantified by reducing the two-dimensional ROC curve into a one-dimensional scalar value called the area under the ROC curve (AUROC). An AUROC is a value between 0 and 1, where 0.5 would lie along the diagonal line and indicate that the model was as effective at classifying samples as random chance. The higher the AUROC, the better the model is at differentiating the classes. Our model robustly distinguished the three cohorts with high accuracy, yielding rounded AUROC values of 1.00 (Fig. 3a), which indicates strong differences in the microbiomes of these cohorts. ICU samples versus healthy or PTLDS samples were correctly classified in 100% of samples, which is to be expected, given the heavy use of antibiotics by such patients that results in severe alteration of the microbiome (39). The model correctly classified 82.4% of PTLDS samples against ICU and healthy controls, with the remaining 17.6% of PTLDS samples being misclassified as healthy (Table 1). We report the relative abundance of the five most important features (OTUs) for sample classification (Fig. 3b). Of note, *Blautia* species (OTU identifiers [IDs] 4474380, 4465907, and 4327141) comprise three of the five most important features for classification and were observed at a significantly greater relative abundance in the PTLDS cohort (8.86% ± 1.26%) than in the ICU (0.070% ± 0.017%) or healthy (1.34% ± 0.18%) cohort (*P* value < 0.0001). Conversely, the two other top five features most important for classification were Staphylococcus aureus (OTU ID 446058), which was present at a significantly higher relative abundance in the ICU cohort (0.95% ± 0.56%) (*P* value < 0.0001) than in the PTLDS (0.0024% ± 0.00030%) or healthy (0.0077% ± 0.0020%) cohort, likely due to it being a widespread nosocomial pathogen (45), and a *Roseburia* species (OTU ID 4481427) elevated in the healthy cohort (0.29% ± 0.050%) compared to PTLDS (0.15% ± 0.045%) (not significant [NS]) or ICU (0.0024% ± 0.0013%) (*P* value < 0.0001) (Fig. 3b).

**TABLE 1:** Probability to distinguish PTLDS from healthy and ICU controls based on the fecal microbiome composition^*a*^

Cohort	Value for cohort		
	ICU	PTLDS	Healthy
ICU	1	0	0
PTLDS	0	0.824	0.176
Healthy	0	0	1

a A random-forest classifier model was used to determine the ability to distinguish PTLDS from healthy and ICU cohorts based on the fecal microbiome. The reported number is the proportion of samples in each cohort classified into a given cohort.

### Elucidation of the effect of antibiotics on patients with PTLDS.

Patients with PTLDS are treated with antibiotics, initially to eradicate B. burgdorferi and often subsequently after treatment failure, which might impact their gut microbiome composition. We therefore examined the possibility that antibiotics alone were responsible for the distinctive microbiomes observed in PTLDS. Out of the 87 patients in the PTLDS cohort, 79 (90.8%) were treated with antibiotics, for Lyme disease and other conditions, within 1 year of sample collection, 64 (73.6%) within 6 months, 36 (41.4%) within a month, and 23 (26.4%) within a week (Table 2). Doxycycline and amoxicillin, both commonly prescribed for the treatment of acute Lyme disease, were the most common antibiotics taken. Note that the patients misidentified as “healthy” in the classifier model (Table 1) received an antibiotic regimen that was no different from that of the bulk of PTLDS patients.

**TABLE 2:** Summation of antibiotic use within the PTLDS cohort^*a*^

Time	No. (%) for:		
	Antibiotic use	Doxycycline	Amoxicillin
1 wk	23 (26.4)	6 (6.9)	12 (13.8)
1 mo	36 (41.4)	12 (13.8)	15 (17.2)
6 mo	64 (73.6)	35 (40.2)	19 (21.8)
1 yr	79 (90.8)	46 (52.9)	24 (27.6)

a Time refers to the period within which antibiotics were taken prior to sample donation. Antibiotic use is the total number of patients (percent) who have used antibiotics within the time frame. Doxycycline and amoxicillin columns describe the number and percentage of PTLDS patients who have taken doxycycline and/or amoxicillin during the indicated time frame.

**FIG 4:**
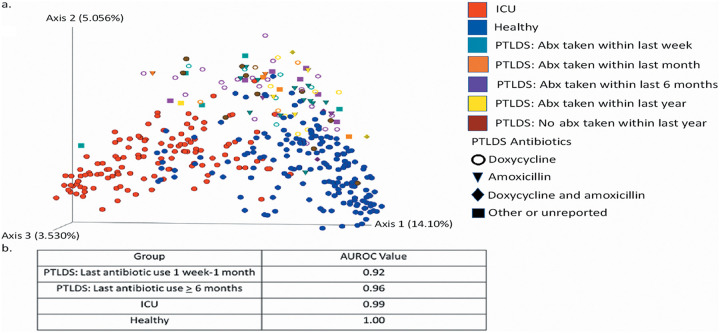
(a) Principal-coordinate analysis of unweighted UniFrac distances of 16S rRNA genes. All samples from patients in the ICU (red) and IT-Healthy and AGP Healthy (blue) cohorts are represented. The time since antibiotic (Abx) treatment before sample collection is indicated by symbol color for PTLDS samples. The most recently taken type of antibiotic taken within 1 week to 1 year of sample collection is indicated by the symbol shape for PTLDS samples. (b) Area under the receiver operating characteristic curve (AUROC) evaluating the ability of a random-forest classifier model to classify the fecal microbiome in the healthy, ICU, and PTLDS cohorts, separated into two groups based on antibiotic use within the last 1 week to 1 month or equal to or greater than 6 months.

To investigate the role antibiotics may play in shaping the microbiome of patients with PTLDS, we used principal-coordinate analysis and identified the type of antibiotic used and the time since last antibiotic use relative to when the stool sample was collected. Importantly, patients with PTLDS did not cluster by time since antibiotic treatment (1 week, 1 month, 6 months, 1 year, or over 1 year) or by type of antibiotic (doxycycline, amoxicillin, both, other, or none) in principal-coordinates analysis (Fig. 4a). We then separated the PTLDS cohort into groups based on how recently a patient had taken antibiotics, within 1 week to 1 month or ≥6 months, and used a supervised random-forest classifier model to evaluate the ability of antibiotic history to distinguish these groups within the PTLDS cohort and healthy and ICU samples. The difference in antibiotic administration regimens did not distinguish patients within the PTLDS cohort (Fig. 4b). Therefore, while antibiotics likely affect the microbiome, our results suggest that antibiotic influence alone cannot explain the distinctive microbiomes of PTLDS patients.

### Grouping patients with PTLDS.

**FIG 5:**
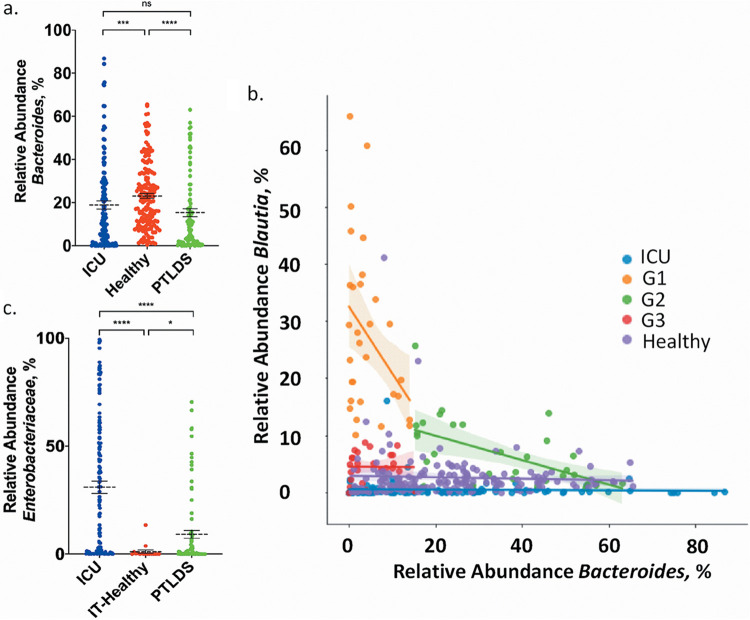
(a) Relative abundances of *Bacteroides* in the fecal microbiomes of healthy and ICU control cohorts and patients with PTLDS. Bars indicate the mean plus or minus the standard error of the mean. Statistical significance was determined using the Kruskal-Wallis (nonparametric) test followed by Dunn’s multiple comparison (****, *P* value < 0.0001; ***, *P* value < 0.001; *, *P* value < 0.01). (b) Relative abundances of *Blautia* versus *Bacteroides* in the fecal microbiomes of healthy and ICU control cohorts and of patients with PTLDS separated into three groups, G1, G2, and G3. The groups were determined based on the relative abundances of *Blautia* and *Bacteroides*: G1, >10% *Blautia* and <15% *Bacteroides*; G2, >15% *Bacteroides*; and G3, <10% *Blautia* and <15% *Bacteroides*. (c) Relative abundances of *Enterobacteriaceae* in the ICU and PTLDS cohorts and the Ion Torrent subset of the healthy control cohort.

While the classifier model indicates a predictive signature of the fecal microbiome in PTLDS, the model misclassifies PTLDS for healthy samples 17.6% of the time (Table 1), suggesting that the microbiome may not be affected in all patients. Thus, we reasoned that subgrouping patients with PTLDS based on important taxonomic features, rather than averaging the entire cohort, would allow us to further identify important aspects of the microbiome in PTLDS. As *Blautia* was the most represented genus in the important features for classification (Fig. 3b), we used *Blautia* as a metric for the groupings. We observed that patients with a relative abundance of *Blautia* over 10% tended to have a decreased abundance of the genus *Bacteroides*, below 15%, compared to an average relative abundance of 23.15% in the healthy cohort (Fig. 5a). Given this, together with the known importance of *Bacteroides* as a common gut symbiont (46, 47) and the correlation between decreased *Bacteroides* in diseases with symptoms overlapping those of PTLDS, such as depression (48), we used *Bacteroides* as our secondary grouping metric. Plotting the relative abundance of *Bacteroides* versus the relative abundance of *Blautia* yielded three distinct subgroups in the PTLDS cohort, which we defined as follows: group 1 (G1), >10% *Blautia* and <15% *Bacteroides*; group 2 (G2), >15% *Bacteroides*; and group 3 (G3), <10% *Blautia* and <15% *Bacteroides* (Fig. 5b). Few samples in the healthy (1.78% of samples) and ICU (0.813%) cohorts overlapped with G1 (high *Blautia* and low *Bacteroides*), which comprised 30.92% of PTLDS samples, but greater overlap existed in G2 and G3 (Fig. 5b). In the classifier model (Fig. 3a), all samples in the test set that were defined as G1 were correctly classified as PTLDS.

Expansion of proinflammatory *Enterobacteriaceae* is a common feature of disease-associated microbiomes (49), and we therefore examined the relative abundance of this group in patients with PTLDS. Although the family *Enterobacteriaceae* was not in the top 5 most important features for classification of the microbiome in PTLDS, healthy, and ICU cohorts, some patients with PTLDS had exceptionally high levels of *Enterobacteriaceae* compared to the healthy control population at Northeastern University (IT-Healthy). Of the 193 OTUs in the *Enterobacteriaceae* family represented in the combined data sets (PTLDS, ICU, and healthy) in this study, the mean relative abundance of *Enterobacteriaceae* in IT-Healthy was 1.14% (median = 0.0275%), compared to an average relative abundance of 9.20% in PTLDS subjects (median = 0.46%). Approximately one-fifth (19.5%) of patients with PTLDS presented with a relative abundance of *Enterobacteriaceae* over 10%. As expected, ICU patients had a higher average relative abundance of *Enterobacteriaceae* (31.21%) (Fig. 5c).

**FIG 6:**
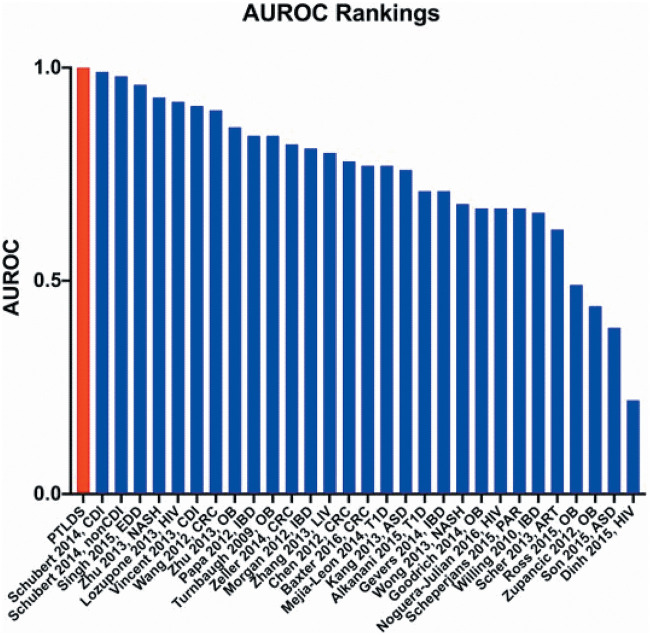
Ranked area under receiver operating characteristic curve (AUROC) reported by Duvallet et al. (50) for the classification of the fecal microbiome in each disease versus a healthy control cohort. ART, arthritis; ASD, autism spectrum disorder; CDI, Clostridium difficile infection; CRC, colorectal cancer; EDD, enteric diarrheal disease; HIV, human immunodeficiency virus; IBD, inflammatory bowel disease; LIV, liver disease; NASH, nonalcoholic steatohepatitis; nonCDI, non-Clostridium difficile infection; OB, obesity; PAR, Parkinson’s disease; T1D, type I diabetes.

Microbiome-associated studies (MAS) have been found to be excellent predictors in various diseases (50), often outperforming genome-wide association studies (31), likely because the microbiome is a confluence of genetics and the environment. ROC analysis of the PTLDS cohort yielded a rounded AUROC of 1.00, correctly classifying patients with PTLDS for 82.4% of samples. These results are similar in accuracy to results for well-established microbiome-associated diseases (50) such as Clostridium difficile infection, while outperforming the predictive capabilities of other MAS, such as inflammatory bowel disease (IBD), in which abnormalities within the microbiome are strongly implicated (Fig. 6).

## DISCUSSION

A conservative estimate of the prevalence of PTLDS in the United States in 2020 is 800,000 (51). The etiopathology of PTLDS is unknown, and the disease lacks suitable laboratory diagnostics and therapeutic methods (5). We aimed to address this ambiguity by investigating the microbiome, which has been shown to play an important role in diseases with symptom overlap with PTLDS. Through 16S rRNA gene sequencing, we identified alterations in the gut microbiome in a cohort of well-characterized patients with PTLDS compared to two healthy control cohorts and an ICU control cohort. The majority of the ICU patients were on antibiotic treatment at the time of collection; this served as a control for antibiotic use among patients with PTLDS. Using a receiver operating characteristic analysis based on a random-forest classifier model, we found that the microbiome of PTLDS patients is distinct from those of ICU patients and healthy controls. *Blautia* species represented three of the five most important features for this classification, and the relative abundance was elevated in the PTLDS cohort compared to those in the healthy and ICU cohorts. Interestingly, an increased relative abundance of *Blautia* has been observed in several other diseases. In patients with type 1 diabetes, elevated *Blautia* abundance was observed and was correlated with increased IA-2 tyrosine phosphatase autoantibodies, important markers of autoimmunity (52). Increased *Blautia* has also been seen in obesity (53), Alzheimer’s disease (54), nonalcoholic fatty liver disease (55), and multiple sclerosis (56).

In addition, approximately one-fifth of patients with PTLDS had a relative abundance of *Enterobacteriaceae* over 10%, while the average relative abundance of *Enterobacteriaceae* in the healthy cohort collected at Northeastern University was 1.14%, consistent with reports of *Enterobacteriaceae* in the healthy gut microbiome (57, 58). Members of the *Enterobacteriaceae* family have a proinflammatory lipopolysaccharide (LPS) in the outer membrane and can exacerbate inflammation (59). A high relative abundance of *Enterobacteriaceae* is reported in inflammatory bowel diseases (60), in metabolic disorders like type 2 diabetes, and in immune diseases like cancers (49). Concomitantly, we report a depletion of *Bacteroides* in the G1 and G3 subsets of the PTLDS cohort which together comprised 64.4% of the PTLDS cohort. *Bacteroides* is a common member of the gut microbiome and plays important, symbiotic roles, such as modulation and regulation of the immune system, maintenance of intestinal integrity, and carbohydrate digestion (46, 47). We have previously reported that γ-aminobutyric acid (GABA) production by human-derived *Bacteroides* is widespread, and there is a correlation between brain signatures of depression and fecal *Bacteroides* levels in patients with major depressive disorder (48). Moreover, *Bacteroides* organisms are major producers of short-chain fatty acids, which have been shown to support host immune homeostasis both locally and systemically (61, 62).

While it is possible given the nature of these aberrations that the microbiome is causal or contributory to PTLDS, establishing this relationship is difficult, as no animal model of PTLDS exists. Our results suggest an intriguing opportunity to test causality by using fecal microbiota transplant (FMT) or defined symbiotic consortia to treat patients with PTLDS. FMT has been successfully used to treat Clostridium difficile infection in patients (63–65). Furthermore, FMT or the administration of symbiotic bacteria has also been shown to be efficacious in treating multiple sclerosis (34), Parkinson’s disease (66), Alzheimer’s disease (67), and rheumatoid arthritis (68) in animal models of disease.

As well as suggesting a potential diagnostic tool through the interrogation of the fecal microbiome, the robustness of these results reinforces the validity of PTLDS by showing strong distinctions between the fecal microbiomes of a rigorously curated cohort of patients with PTLDS, patients in the ICU, and healthy controls. Previously reported biomarkers further validate PTLDS and provide an opportunity for the field to progress. These biomarkers include quantitative immune alterations; patients with PTLDS present with elevated levels of the T-cell chemokine CCL19 compared to patients with acute Lyme disease who have returned to health (15), an increase in the cytokine IL-23 (16), a decrease in the CD57 lymphocyte subset (17, 18), and a decreased plasmablast response prior to treatment (13). Furthermore, a pilot study used [^11^C]DPA-713 PET imaging to study cerebral glial activation and found that several brain regions had higher [^11^C]DPA-713 binding in patients with PTLDS than in healthy controls (12). In addition to these biomarkers, Fallon et al. (69) developed a survey, the General Symptom Questionnaire-30 (GSQ-30), to assess symptom burden and changes; patients with PTLDS reported higher GSQ-30 scores before treatment and maintained these scores until 6 months posttreatment. The GSQ-30 could be a powerful tool to accompany biomarkers like the gut microbiome in PTLDS. The existence of these biomarkers, along with the microbiome signature that we report, contributes to the evidence for a biological basis for PTLDS. It also supports clinical and accumulating research evidence, first published for treatment trials and population-based studies (70–72), that persistent symptoms after treatment of Lyme disease are common and can significantly impact quality of life. The lack of sensitivity of PTLDS symptoms such as fatigue, pain, and cognitive dysfunction can lead to the conclusion that they are not different than the background noise levels in the general population (73). However, studies operationalizing the proposed case definition for PTLDS (11) which utilize standardized symptom and quality-of-life measures have shown that the prevalence and magnitude of these symptoms are often more severe (4, 74).

Antibiotic use likely affected the microbiome of patients with PTLDS; 9.2% of patients with PTLDS were on antibiotic treatment during the time of collection, and many had extensive antibiotic treatment in their recent health history. While antibiotics likely alter the microbiome composition in patients with PTLDS, our data show that the PTLDS microbiome does not cluster by antibiotic history, and having PTLDS is a better classifier than antibiotic history. Therefore, it is unlikely that antibiotic use alone explains the distinct PTLDS microbiome. Importantly, the microbiome of PTLDS patients was distinct from the microbiome of patients in the ICU. Regardless of the cause of the disruption observed in the microbiome in PTLDS, our data suggest that therapeutic intervention targeting the microbiome may ameliorate PTLDS symptoms. In conclusion, we report that a cohort of patients with PTLDS have microbiomes distinct from those of healthy and ICU controls. Furthermore, we show that through machine learning we can use the microbiome as a high-fidelity indicator of PTLDS. We reinforce the validity of this disease by showing strong distinctions between a rigorously curated cohort of patients with PTLDS and controls.

## MATERIALS AND METHODS

### Ethics statement.

The Northeastern University Institutional Review Board (IRB no. 16-02-22 and 08–11-16), Johns Hopkins Medicine Institutional Review Board (IRB no. 00035457), and the University of California San Diego Institutional Review Board (IRB no. 141853X) approved the collection of feces from human subjects. Informed written consent was obtained from all participants.

### Study cohorts and sample collection.

**(i) Posttreatment Lyme disease syndrome cohort.** The posttreatment Lyme disease syndrome (PTLDS) cohort is part of the Study of Lyme disease Immunology and Clinical Events (SLICE) curated at the Johns Hopkins Lyme Disease Research Center. Detailed enrollment and eligibility criteria for this cohort have been previously described (4). Briefly, patients with PTLDS had medical record documentation of prior Lyme disease meeting the CDC surveillance case definitions with appropriate treatment and had current patient-reported symptoms of fatigue, cognitive dysfunction, and/or musculoskeletal pain resulting in functional impairment. Many of those enrolled had received subsequent antibiotics for treatment of their persistent symptoms, and participants were permitted to be actively taking antibiotics for their condition at the time of enrollment. Patients with PTLDS were also excluded for a range of preexisting or comorbid conditions with significant PTLDS symptom overlap and/or immunosuppressive effects. Information on appropriate antibiotic treatment for Lyme disease was abstracted from the medical record; subsequent antibiotic use was recorded as part of the research study visit.

Subjects were provided with stool collection containers containing 9 ml of 20% glycerol and BBL culture swabs (Becton, Dickinson and Company, Sparks, MD). From a single stool sample produced at any time of day, stool was self-collected into the collection container to reach ∼10 ml and swabs were taken; samples were returned to the Johns Hopkins Lyme Disease Research Center (MD) and stored at −80°C. Samples in stool collection containers were sequenced using Ion Torrent technology as described below, and swabs were sequenced using Illumina technology at the Knight Lab at University of California San Diego (UCSD). Additional metadata on prior treatment and current antibiotic use from participants with PTLDS were gathered as part of the larger clinical case series study.

**(ii) Healthy control cohort.** The healthy control cohort consisted of two healthy populations: a healthy cohort at Northeastern University (IT-Healthy; Boston, MA) and 152 donors from a healthy subset of the American Gut Project (38) (AGP Healthy). Sample processing for these cohorts was performed according to Earth Microbiome Project protocols (75). Using stool collection vessels (Medline Industries), one fresh stool sample was self-collected from 17 healthy adult donors. Donors were excluded if they were currently taking antibiotics or if they had taken antibiotics for at least 2 weeks at the time of collection. A sample of the stool was immediately placed in 9 ml of oxygen-prereduced phosphate-buffered saline (PBS) to a total of ∼10 ml of slurry in a 50-ml collection tube (Fisher Scientific). The stool slurry was quickly homogenized in a Coy anaerobic vinyl chamber (Coy Laboratory Products, Inc.) in 5% hydrogen, 10% CO_2_, and 85% nitrogen at 37°C. Samples were stored at −80°C and sequenced using Ion Torrent technology as described below. A healthy subset of the American Gut Project was identified as previously described (38); 152 samples were randomly selected from the healthy subset. Samples were collected and sequenced as previously reported (38).

**(iii) ICU cohort.** The ICU cohort consists of 123 samples from two time points (within 48 h of ICU admission and at ICU discharge or on ICU day 10) from critically ill patients in the intensive care unit in four centers across the United States and Canada as reported previously (39). Sample collection and processing for this cohort were performed according to Earth Microbiome Project protocols (75). This cohort served as a control for the effect of antibiotics on the microbiome, as the ICU patients had omnipresent antibiotic use. All ICU patients were treated with differing antibiotic regimens.

### Preparation of DNA and 16S rRNA sequencing protocols.

**(i) Ion Torrent sequencing.** DNA extraction and sequencing were performed by MR DNA (Shallowater, TX) on an Ion Torrent PGM. The V4 variable region was amplified using PCR primers 515/806 (515F, GTGCCAGCMGCCGCGGTAA, and 806R, GGACTACVSGGGTATCTAAT) in a single-step 30 cycle PCR with the HotStarTaq Plus master mix kit (Qiagen, USA). The following conditions were used: 94°C for 3 min and 30 cycles of 94°C for 30 s, 53°C for 40 s, and 72°C for 1 min, followed by a final elongation step at 72°C for 5 min.

**(ii) Illumina sequencing.** Using the primers 515f/806rB, the V4 region was amplified and was sequenced as previously described (76) using an Illumina MiSeq (Knight Lab, UCSD). Sequencing data for the ICU cohort and the American Gut project were obtained in Qiita (40) (study IDs 2136 and 10317).

### 16S rRNA data analysis.

Raw sequencing data were uploaded and processed in Qiita (40) (study ID 11673); the sequences were demultiplexed and trimmed to 150 bp, and closed-reference OTUs were picked with Greengenes 13-8 (77) on an OTU similarity level of 97%. The OTU table was rarefied to 10,000 reads. Data were subsequently analyzed using the software package QIIME2 (41). Since Ion Torrent and Illumina sequencing followed the Earth Microbiome Project protocol, the sequencing platform did not have a measurable effect on the data (Fig. 1) and we were able to combine the sequencing platforms for analysis. To assess the ability of the PTLDS microbiome to be distinguished from healthy and ICU controls, the sample classifier tool in QIIME2 was used (43). A random-forest classifier was trained and evaluated. ROC curves were generated to summarize the true- versus false-positive rates; the area under the curve was calculated and reflects the ability of the classifier to distinguish between cohorts. The top five most important features for distinguishing the microbiomes were reported.

### Data availability.

Data generated in this study are available in Qiita (https://qiita.ucsd.edu/study/description/11673) and the European Bioinformatics Institute (https://www.ebi.ac.uk/ena/browser/view/ERP122507).
