# Supplementary figures and images for: A Distinct Microbiome Signature in Posttreatment Lyme Disease Patients
Source: mBio. 2020 Sep 29;11(5):e02310-20. doi: 10.1128/mBio.02310-20 (PMC7527730; doi:10.1128/mBio.02310-20)

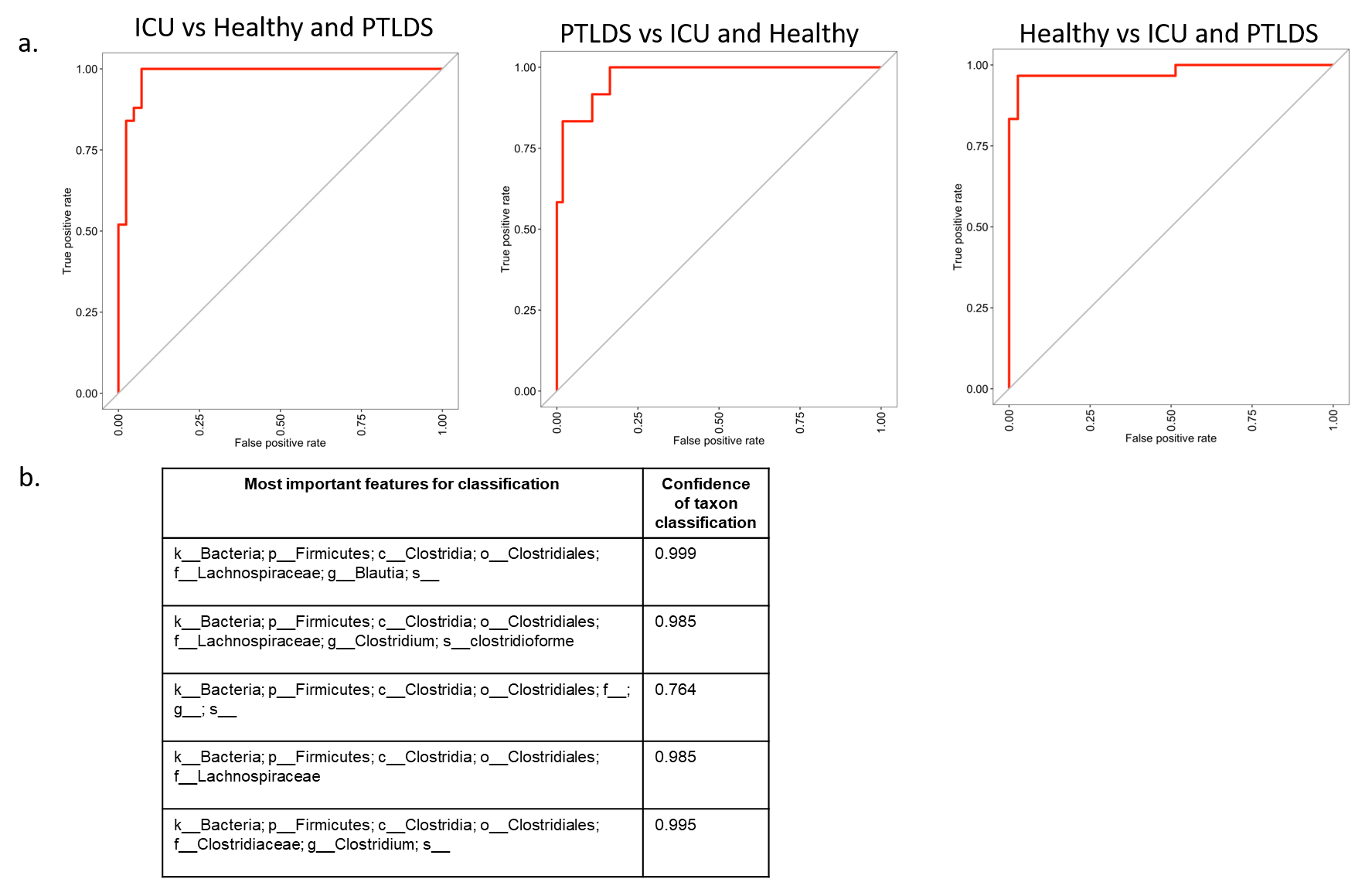

Supplement: FIG S1 [file mBio.02310-20-sf001.tif]
